# Isolation and Quantification of Polystyrene Nanoplastics in Tissues by Low Pressure Size Exclusion Chromatography

**DOI:** 10.3390/jox12020010

**Published:** 2022-05-09

**Authors:** François Gagné

**Affiliations:** Aquatic Contaminants Research Division, Environment and Climate Change Canada, Montréal, QC H2Y 2E7, Canada; francois.gagne@ec.gc.ca

**Keywords:** polystyrene, nanoparticle, size exclusion chromatography, mussels

## Abstract

Ecotoxicity investigations of plastic nanoparticles (NPs) should pay more attention to their ability to pass barriers, accumulate, and initiate toxicity in cells. The purpose of this study was to develop a simple size exclusion chromatography (SEC) methodology to measure plastic NPs in biological tissues. A SEC column was prepared using a high-resolution gel for large macromolecules to separate plastic NPs from the protein/lipid pools in tissues. It was necessary to prepare the samples in high salt and non-ionic detergent (0.5 M NaCl and 0.2% Tween-20) and apply 0.2% Tween-20 containing 14 mM NaCl for the elution buffer to limit proteins adsorption to NPs. This methodology was able to resolve 50 and 100 nm polystyrene NPs from the protein/lipid pools in tissue homogenates. The fluorescent dye neutral red (NR) was also used for transparent NPs. Moreover, a sample fractionation step was also proposed for plastic NPs concentration using a salting-out methodology with saturated NaCl (5 M) and acetonitrile. Polystyrene NPs partition in acetonitrile, which were further analyzed by SEC. This methodology was tested in two case studies with clams collected in a high boat traffic (harbor) area and with caged freshwater mussels downstream of a large urban area. Although the present methodology was developed with polystyrene NPs it should be amenable to other plastic polymers that react with the NR fluorescent probe.

## 1. Introduction

The observation that plastic materials are found at a global scale represents a major contamination problem, thus raising concerns about their long-term impacts on ecosystems. Indeed, plastic wastes reach millions of tons per year, and not all are recycled back into our economy, contaminating both terrestrial and aquatic environments. Microplastics (5 mm to 1 µm) are found in consumer products such as cosmetics, find their way into wastewater treatment plants—ill equipped to remove them—and are released into the aquatic environment [1,2]. To make this situation worse, the environment breakdown of plastics materials by abiotic and biotic processes will release an exponential number of particles as the size decreases, reaching the nanoscales (<100 nm). Plastic nanoparticles (NPs) are defined as particles with sizes generally <100 nm but some include the 100–1000 nm range as well. Small NPs (<100 nm) can become readily bioavailable towards organisms and are absorbed in cells [3,4].

Plastic NPs are considered toxic for organisms due to their ability to bioaccumulate in tissues, even passing the blood–brain barrier, leading to oxidative stress and tissue damage [5,6]. A recent meta-analysis on plastic NPs revealed that these NPs significantly decrease survival, and disrupt the behavior and reproduction of fish and invertebrates [7]. NPs are also known to produce oxidative stress and series of biophysical changes (viscosity and altered fractal organization of proteins/enzymes) in aquatic organisms, leading to toxicity [8,9]. Plastics NPs could also act as vectors by absorbing existing toxic compounds such as bisphenol A, polyaromatic hydrocarbons, and heavy metals [10]. This could modify the bioavailability and exacerbate the toxic impacts of these existing environmental pollutants. In addition to oxidative stress, polystyrene NPs were also genotoxic to *Mytilus galloprovincialis* mussels, most notably in gills [11], suggesting long-term negative effects. Methods to quantify plastic NPs in tissues are scarce, which limits our understanding about their occurrence in the environment, distribution in tissues, and health impacts. Plastics are readily stained by the solvatochromic dye, neutral red (NR), often used to detect the presence of both micro and plastic NPs [6,11]. However, NR also reacts to nonpolar lipids in cells, which could interfere with the assay. Hence, there is a need to find other means to determine plastic NPs in biological tissues involving hyphenated procedures. More recently, a more specific fluorescent assay for polystyrene NPs in tissues was proposed using molecular rotor probes [12,13]. The presence of plastic NPs in the crowded environment of cells could disrupt the protein networks and lipid dynamics producing organized (nematic-like) liquid crystals, protein condensation/denaturation, and changes in metabolic pathways [8]. A methodology to study the interactions of plastic NPs in cells with respect to proteins and lipids would be valuable to help us better understand the uptake and toxicity of NP at the molecular level.

The purpose of this study was therefore to develop a low-pressure size exclusion chromatography (SEC) methodology for polystyrene NPs in tissue homogenate fraction. The method should be available to resolve NPs from the protein and lipids pool in the cytoplasm and permit some degree of quantification. The proposed method was first developed with fluorescently labeled polystyrene NPs, albumin, and in spiked homogenate fractions. The resulting method was optimized to analyze plastic NPs directly in marine and freshwater bivalve tissues in two real-life case studies.

## 2. Materials and Methods

### 2.1. Sample Preparation

Tissue samples were dissected on ice, weighted and placed in three volumes of 140 mM NaCl containing 10 mM Hepes-NaOH pH 7.4, 1 mM EDTA and 1 mM dithiothreitol. The tissues were homogenized using a Teflon pestle tissue grinder (5 passes) and centrifuged at 3000× *g* for 20 min. The supernatant was collected, filtered on the 0.45 µm pore membrane and stored at −85 °C until analysis. A fractionation procedure was also studied based on salting-out methodology. The homogenate sample (300 µL) was mixed with 400 µL of saturated NaCl (5 M) and 300 µL of 100% acetonitrile (ACN) for 10 min and centrifuged at 3000× *g* for 5 min. Polystyrene NPs readily partitions in the upper ACN layer and was collected for chromatographic analysis.

### 2.2. Size Exclusion Chromatography

Plastic NPs were analyzed by low-pressure size exclusion chromatography using the Bio-logic system (Biorad, Ontario, Canada). The column (40 cm × 1 cm) was filled with Sephacryl S-500 gel and equilibrated with 0.2% Tween-20 and 14 mM NaCl at pH 7.4 at a flow rate of 1 mL/min. Sephacryl S-500 is a high-resolution gel designed for the separation of large macromolecules between 2 × 10^7^ to 4 × 10^4^ g/mole size range and suitable to resolve plastic NPs from the protein/lipid pool. Conductivity (NaCl and other salts) and absorbance at 280 nm (proteins) were continuously monitored. One mL fractions (0.75 mL/min) were collected for NR staining (10 µM final concentration). Clear and fluorescently labeled (yellow–green Fluoresbrite) polystyrene nanoparticles of 50 and 100 nm were purchased at Polyscience Inc. (Warrington, PA, USA) and were used for column calibration along with NaCl and albumin (66 kDa).

Calibration was achieved using fluorescently labeled polystyrene NPs in the absence/presence of biological sample (proteins) to determine the optimal separation conditions. As plastic NPs tend to adsorb proteins and lipids, forming a corona at the surface, various loading and elution buffers were tested to find optimal conditions at the levels of detergent type (ionic vs. nonionic detergents), concentration, solvent (acetonitrile, acetone, and ethanol) and salt concentration (NaCl). It was found that the addition of albumin and tissue homogenate extracts (proteins/lipids) interacted with nanoparticles in low salt (<10 mM NaCl) and detergent concentrations (<0.05%). Incubating the homogenates fractions in high salts (0.7 M NaCl) in the presence of nonionic detergent (0.05–0.2% Tween-20) in the sample buffer and nonionic detergent (0.2% Tween-20 in 14 mM NaCl) for the elution buffer eliminated nanoparticle–protein interactions and facilitated separation of plastic NPs from the proteins/lipids pool.

Homogenate fractions were either directly injected in the column or previously fractionated by a salting-out methodology using saturated NaCl/ACN. For the direct injection, the homogenate supernatants (3000× *g* 10 min) were thawed on ice and mixed with one volume of 1.4 M NaCl and 0.2% Tween-20 buffered at pH 7.4 (with 1 M K_2_HPO_4_) for 5 min. As explained above, the addition of salts and nonionic detergents were necessary to keep proteins from adhering to the nanoparticle’s surface and to exclude suspended plastic particles (density). For the salting-out step, 300 µL of the homogenate was mixed with 400 µL of 5 M NaCl and 400 µL of ACN. The sample was mixed and centrifuged at 3000× *g* for 10 min as described above. The ACN upper phase was mixed with 0.1 volume of 1.4 M NaCl/Tween-20 0.1% and injected to the chromatography column (0.25 mL) using the same elution buffer (0.2% Tween-20, 14 mM NaCl). A volume of 0.25 mL of transparent or fluorescent plastic nanoparticles (50 and 100 nm), albumin (66 kDa), and 0.5 M NaCl (total volume) was also injected in the column for calibration. The flow rate was 0.75 mL/min and 1 mL fractions were collected to a total volume of 30 mL. The absorbance at 280 nm (proteins and polystyrene) and conductivity (mS) were continuously measured.

The collected fractions were stained with 10 µM NR dye (from a 100 µM stock solution prepared in 0.2% Tween-20 and 10% ethanol in 14 mM NaCl) and fluorescence was measured at 485 nm excitation and 530 nm (40 nm bandpass) (Turner Biosystems, Sunnyvale, CA, USA). The blank consisted of the elution buffer, and increasing concentrations (0.05–1 µg/mL) of 50 nm diameter transparent polystyrene NPs (Polyscience Inc., Warrington, PA, USA) were used for calibration. The elution volume of the analyte (Ve) was determined based on NR fluorescence, and total volume (Vt) of the column was determined by NaCl conductivity spike from the sample buffer. The void volume (Vo) was based on the supplier information (around 24% of the total column volume = 7.5 mL). The elution profiles data were expressed as Ve/Vt, which permits comparisons with other column volumes.

### 2.3. Case Studies

The proposed methodology was used to screen for plastic NPs in two real-life case studies. The first case study consisted of sampling wild *Mya arenaria* clam populations from a clean or reference site in the Saint-Lawrence estuary (Baie du Moulin à Baude, Québec, Canada) and polluted site (harbor) at Baie Sainte Catherine supporting regular boat tourism activities (Québec, Canada). The polluted site was located some 8 km south from the reference site in the Saint-Lawrence River estuary. A total of 30 clams between 4–8 cm longitudinal shell length were collected by hand and frozen at −20 °C for transportation to the laboratory. At the laboratory, the clams (*N* = 4) were thawed on ice and the soft tissues homogenized in a tissue grinder apparatus in ice-cold homogenization buffer as described above. The homogenates were analyzed by chromatography in each individual (homogenates were not pooled). The second case study consisted of experimentally caged *Elliptio complanata* mussels collected at a pristine lake (Lac des Écorces, Québec, Canada) and 5 km downstream of a municipal wastewater dispersion plume of a largely populated city (population of 1.8 million, City of Montréal, Québec, Canada). The mussels (mean size of 6 ± 0.5 cm, *N* = 10 per cage) were exposed for 3 months during the summer of 2017 (July to September) in cages consisting of 1 × 0.25 m cylindered nets fixed on cement blocks (5–10 kg) and immersed between 2–3 m depth. At the end of the exposure period, the mussels were processed in the same way as the clams, with the exception that the digestive glands of mussels were used instead of whole tissues and kept at −80 °C.

### 2.4. Data Analysis

Sample preparation and column optimisation experiments were repeated three times and the mean value with standard deviation was used as the descriptive statistics. The chromatographic elution profiles in absorbance, conductivity, and NR fluorescence data were normalized at 1 to facilitate data visualization and reporting: (sample reading—blank (elution buffer))/(maximal reading—blank). A number of four individuals were collected for *Mya arenaria* clams and *Elliptio complanata* caging experiments for the chromatography analyses with either direct injection or after NaCl/ACN fractionation. Graphical and statistical analysis were performed using the SYSTAT software package (version 13).

## 3. Results and Discussion

**Figure 1:**
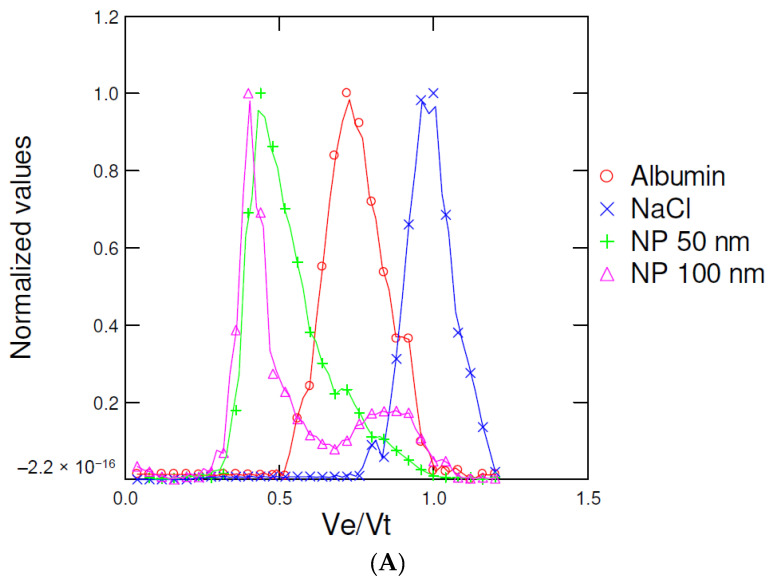

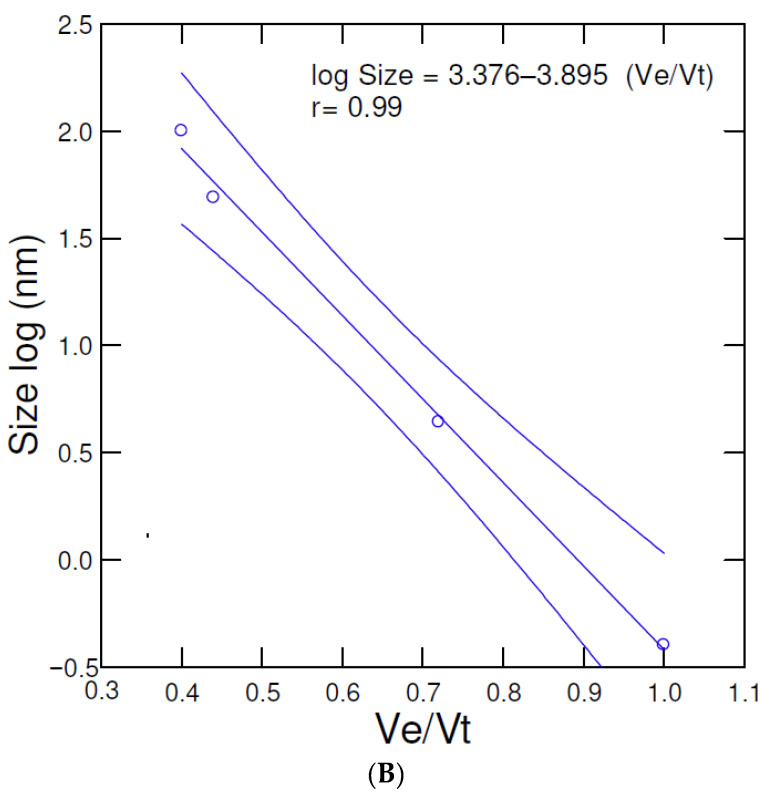
Gel chromatographic analysis of fluorescently labeled plastic NPs. Elution profile of polystyrene NPs (**A**) using 100 nm, 50 nm polystyrene NPs (fluorescence), albumin (absorbance 280 nm, MW = 60 kDa), and NaCl (conductivity: mS); calibration of the size exclusion chromatography column (**B**).

The proposed methodology makes use of a high-resolution gel (Sephacryl S500) designed to resolve large macromolecules between 20 million and 400,000 daltons (g/mol). The upper protein size range is in the order of circa 750 kDa containing many thousands of amino acids, thus the gel matrix should be able to resolve plastic NPs larger from the protein/lipid pool in cells. Since SEC separates particles based on the Stokes radius rather than weight, the following discussion will be provided in size rather than mass. It is estimated that the protein size range is between 3 and 700 kDa where large 500 kDa globular proteins theoretically occupy a radius of 5.2 nm, which is well within the separation range of the gel matrix [14] and sets the theoretical lower size range of the column. Hence, the gel matrix offers the possibility to separate large plastic nanoparticles (10–1000 nm) from the protein pool in the sample without the need to extract/purify nanoparticles before running the column (Table 1). Large filamentous proteins (e.g., actinomyosin) should precipitate during centrifugation with appropriate speeds depending of the tissues. In the present study with molluscs, homogenisation with blender or Teflon pestle followed by 3000× *g* centrifugation appeared satisfactory. To validate these assumptions, the reported diameter of albumin is about 4 nm (mass of 66 kDa) [15] and should separate well from 50 or 100 nm NPs. A 40 cm × 1 cm column was filled with Sephacryl S-500 gel for separation of pure fluorescently labeled polystyrene nanoplastics (50 and 100 nm mean diameter), albumin, and NaCl (Figure 1). The total volume (Vt = 25 mL) was calculated with a conductivity (NaCl) peak, which permeates completely the gel beads, and the theoretical void volume (Vo) of the column was at Vo/Vt = 0.3 according to the supplier’s information for Sephacryl S500 (Table 1). The 100 and 50 nm NPs were resolved at Ve/Vt = 0.4 and 0.45 respectively, albumin eluted at Ve/Vt of 0.75, and NaCl at Ve/Vt = 1 using the 0.2% Tween-20–14 mM NaCl elution buffer. The addition of the non-denaturing detergent (Tween-20) was necessary to maintain separation of albumin from nonspecific hydrophobic interactions of polystyrene NPs. Indeed, NPs adsorb proteins forming a corona [16] and could change the elution volume (Ve) for polystyrene NPs. This was observed by lowering the detergent concentration to 0.01%, which increased the Ve/Vo to 0.60 for the fluorescent NPs in the presence of 1 mg/mL albumin (results not shown). The addition of denaturing detergent such as SDS (0.1%) denatured albumin and eluted at the void volume Vo/Vt = 0.30 and overlapped upper size range of plastic nanoparticles (>100 nm). A significant and linear relationship between the size (log size in nm) and the Ve/Vt of the samples was obtained with r = 0.99 (Figure 1B). This relationship sets the upper and lower size range of NPs between 161–11 nm respectively.

**Figure 2:**
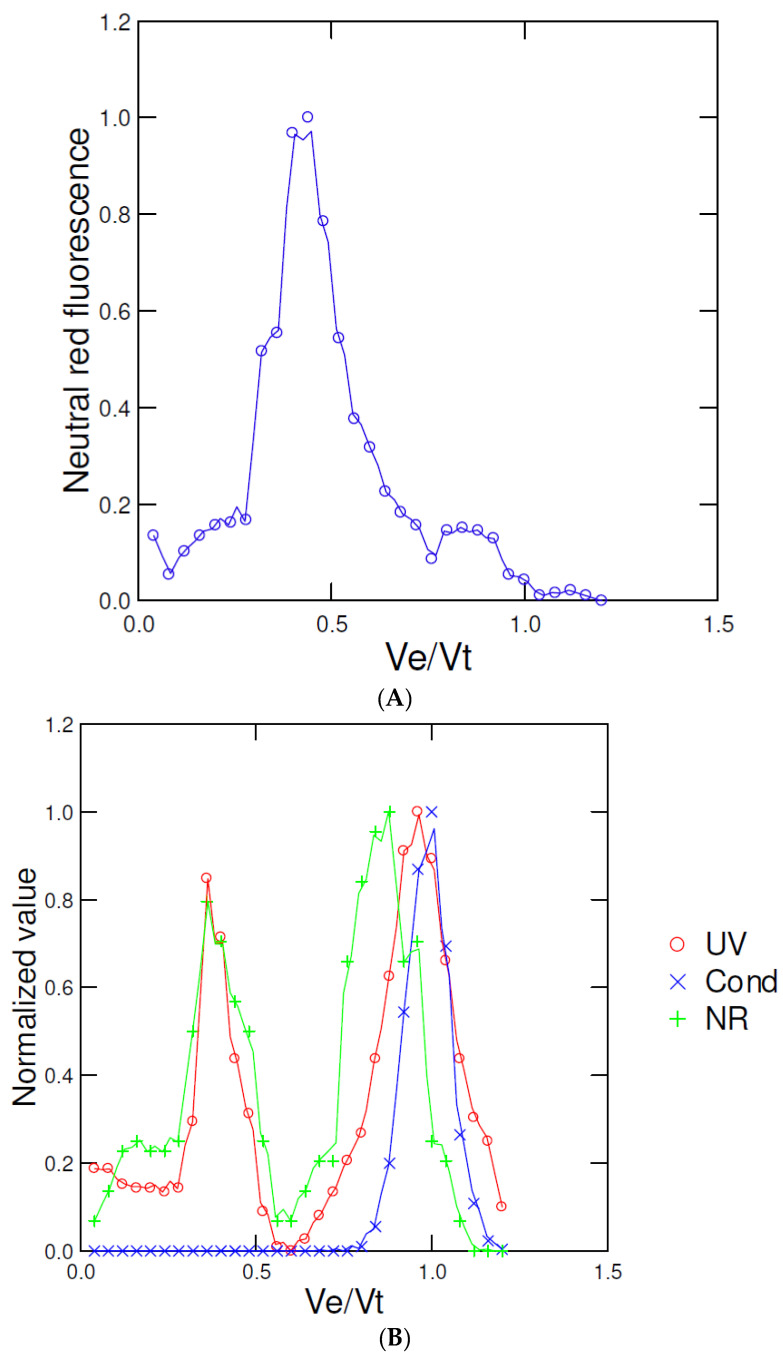
NP addition in mussel tissue homogenates. Pure transparent 50 nm polystyrene NPs at 10 µg/mL (**A**) and spiked mussel tissue homogenates (**B**), centrifuged at 3000× *g* for 10 min, the supernatant mixed with one volume of 1.5 M NaCl containing 0.1% Tween-20 and injected into the column. The eluted samples (1 mL) were collected and were analyzed for absorbance at 280 nm, conductivity and neutral red (NR) staining.

We tested this chromatographic method with transparent polystyrene NPs (50 nm diameter) alone and in *Mya arenaria* soft tissues homogenates (3000× *g* supernatant) using NR staining for plastic particles (Figure 2). The solvatochromic NR dye is well-recognized as a fluorescent dye for plastics but also stains lipids [12,17]. Transparent polystyrene NPs eluted at Ve/Vt = 0.45 based on NR fluorescence (Figure 2A). The addition of soft tissues fractions revealed two peaks at 280 nm (Figure 2B): one at Ve/Vo corresponding to polystyrene NPs and the other at Ve/Vo = 0.9–0.95 corresponding to the protein/lipids pool. NR staining revealed also two major peaks overlapping with 280 nm absorbance corresponding to polystyrene NPs at Ve/Vo = 0.44 and the protein/lipid pool at Ve/Vo = 0.9. The elution profile of tissue fractions alone did not show any peaks at Ve/Vo < 0.7 in keeping with the resolution range of the gel matrix. The NR dye is a well-recognized fluorescent stain for many types of plastics such as polyethylene, polystyrene, nylon, polyethylene terephthalate, and polypropylene at the micro- and nanoscales, thus could be used with these plastic polymers [12,18]. The average length of phospholipids is about 2 nm [19] giving a Ve/Vo = 0.8–0.9 overlapping to the second NR peak, hence not suitable for measuring small plastic NPs (<10 nm). The presence of the detergent Tween-20 in the elution buffer also serves to solubilize membranes vesicles in the extracts removing larger lipid vesicles/structures in the sample.

**Figure 3:**
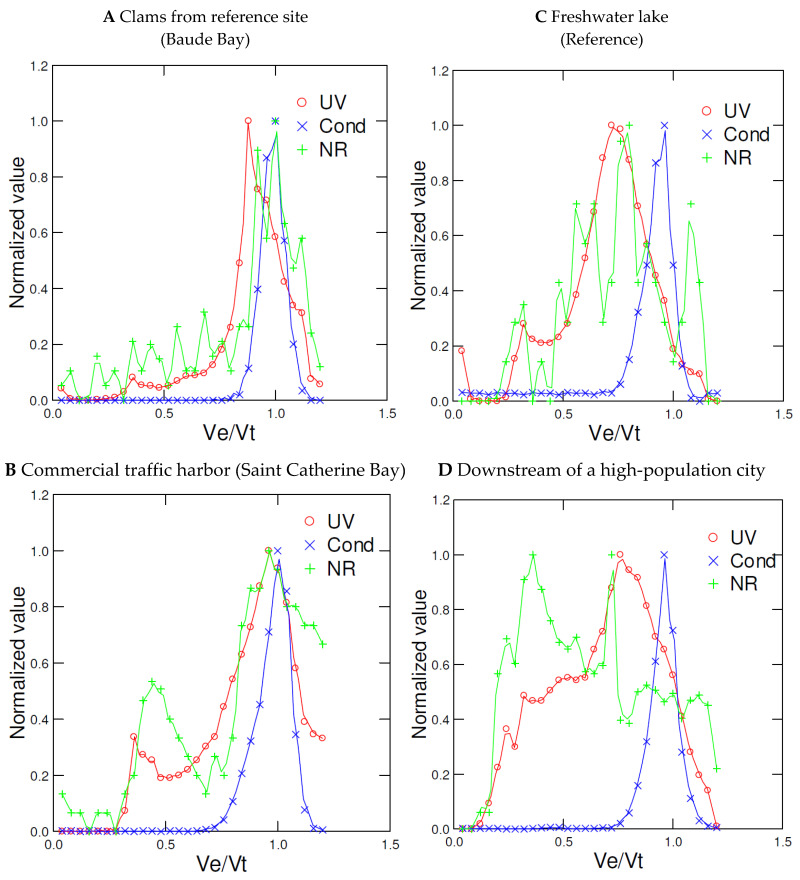
Chromatographic profile marine and freshwater bivalves exposed to anthropogenic pollution. Representative chromatographic profiles of *Mya arenaria* clam tissue homogenates from a reference site (**A**), a harbor supporting intense boat traffic (**B**), *Elliptio complanata* mussel digestive glands caged at reference lake (**C**), and downstream of a large-population city (**D**) for 3 months during the summer of 2017. The homogenates were centrifuged at 3000× *g* for 10 min, the supernatant mixed with one volume of 1.5 M NaCl-0.1% Tween-20 and directly injected to the size exclusion chromatography column. The data represented the measurements at each 1 mL fraction and normalized to 1 for viewing.

This chromatographic procedure was used to screen for plastic NPs in two real-life case studies, one involving wild *Mya arenaria* clams collected at a harbor/marina and the second with caged *Elliptio complanata* mussels at downstream sites of a major urban area, both generally associated to plastics pollution [20]. The first case consisted of feral *Mya arenaria* clams collected at a reference site under no direct source of pollution and a harbour site supporting intense commercial and touristic boating activities (Figure 3A,B). Clam tissues collected at the reference site showed one major UV absorbance (280 nm) peak at Ve/Vt = 0.8 corresponding to the protein pool, one NR peak at Ve/Vt = 0.8–0.9 corresponding to hydrophobic proteins and lipids, and a conductivity peak at Ve/Vt = 1 corresponding to salts/NaCl (Figure 3A). Clams collected at the contaminated harbor site showed two major UV and NR peaks at Ve/Vo = 0.4 and 0.8 (Figure 3B) corresponding to large-size compounds consistent with UV-absorbing plastics (polystyrene and polyethylene terephthalate) followed by the protein/lipid pool peak. Large hydrophilic carbohydrate-based polymers (sugars, glycogen), if present, do not absorb at 280 nm and should not interact with the NR dye. The second case concerned exposure of caged mussels exposed to pristine lake (reference) and downstream of a highly populated city of circa 3 million inhabitants in the Saint-Lawrence river (Figure 3C,D). Sediments in the Saint-Lawrence river were recently reported contaminated by microplastics [20] and prompted investigation on plastic NPs in this study. The major form of plastic was polyethylene (UV absorbance negative and NR positive) and reached densities of 1.4 × 10^5^ microbeads·m^−2^. *Elliptio complanata* digestive gland homogenates caged at the reference lake revealed one major band at Ve/Vt = 0.75 and one small band at the void volume (Figure 3C). One conductivity peak was observed at the Ve/Vt = 1 corresponding to dissolved salts with no evidence of conducting large-size molecules (i.e., absence of peaks at Ve/Vt < 0.8). The NR fluorescence peak generally followed the UV peak with a major band at Ve/Vt = 0.8 of the protein/lipid pool. In mussels caged downstream of a large city, the UV signal was distributed over the elution profile (Figure 3D) with a maxima at Ve/Vt = 0.75. The same conductivity peak at Ve/Vo = 1 was obtained, suggesting no presence of conductive large-molecular-size compounds (e.g., elemental nanoparticles). In the case of NR staining, we observed two major peaks at Ve/Vt = 0.6–0.65 and Ve/Vt = 0.35–0.5, corresponding to compounds of sizes ranging from 11 to 120 nm. This suggests that exposure of mussels to urban activities leads to a complex distribution pattern of NR-stained materials outside the protein and lipids pools in tissues. This is in keeping with the reported levels of microplastics in sediments in the Saint-Lawrence river [20].

**Figure 4:**
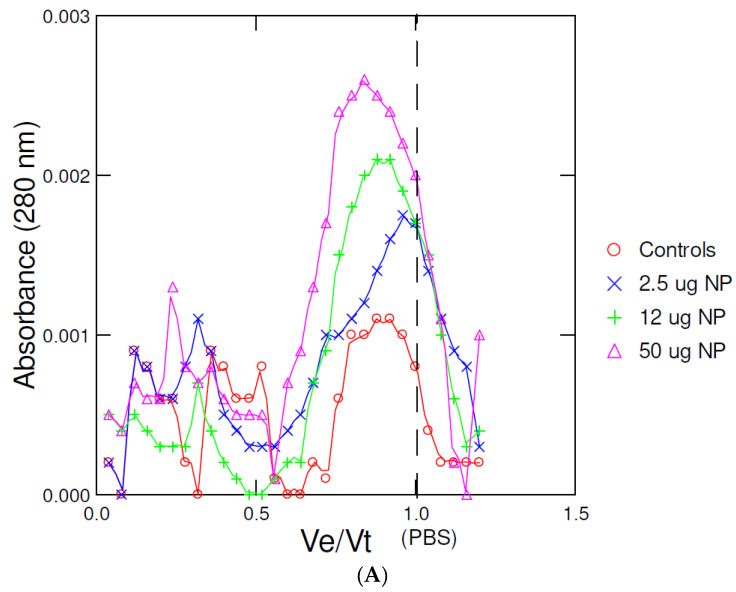

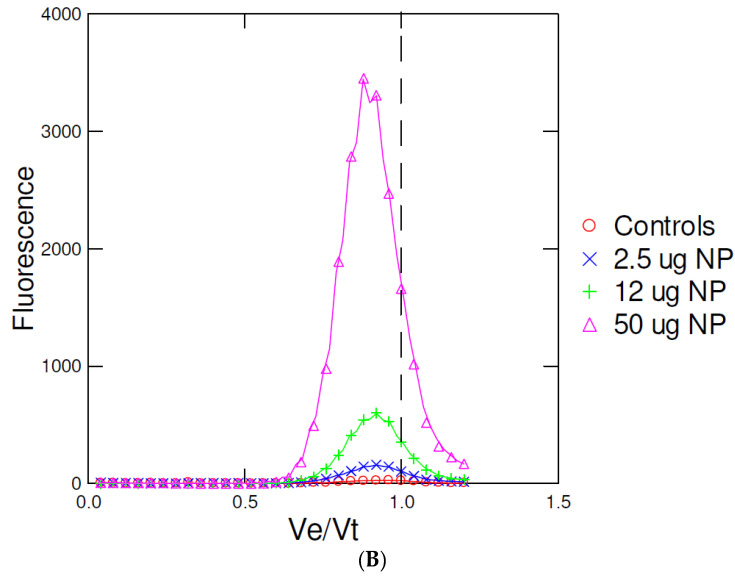
Calibration of the NaCl/acetonitrile fractionation method. Mussel homogenates were spiked with increasing quantities of fluorescently-labeled polystyrene NPs, fractionated using the salting-out step, and the acetonitrile upper phase injected in the SEC column. The elution buffer consisted of 0.2% tween-20 and 14 mM NaCl at pH 7.4. Absorbance at 280 nm (**A**) and fluorescence (**B**) were taken at each 1 mL volume of the eluate. The flow rate was 0.75 mL/min.

Direct sample analysis of homogenate fractions offers the advantage of measuring other endpoints (enzyme activities, proteins) or specific metabolites in addition to the presence of nanoplastic materials. In the attempt to increase the sensitivity of this methodology and as a means to remove potential interfering large protein filaments, a salting-out step with saturated NaCl and acetonitrile (NaCl/ACN) was used to extract and concentrate NPs. Mussel homogenates were spiked with increasing amounts of fluorescent polystyrene NPs, fractionated with NaCl/ACN methodology, and centrifuged at 3000× *g* for 5 min. The ACN upper phase was mixed with 0.1 volume of 1.4 M NaCl (for total volume calibration of the column) and resolved by SEC with the same elution buffer. Because polystyrene absorbs at 280 nm, the absorbance readings of the elution profile were included (Figure 4A). The data revealed that UV absorbance increased linearly but at Ve/Vt = 0.75–0.8 with the added NPs. In an unspiked sample, low absorbance at 280 nm was measured at Ve/Vt = 0.95–1, suggesting the presence of nonprotein UV-absorbing compounds—perhaps nonpolar metabolites such as tyrosine or phenylalanine amino acids/peptides. Lipids would elute near this volume but would not absorb at 280 nm. The fluorescence peak of the fluorescent NPs eluted at Ve/Vt = 0.75 and peak height/area increased linearly with added NP in the homogenates (Figure 4B). The elution of polystyrene at these fractions (Ve/Vt = 0.75–0.8) suggests that polystyrene NPs shrunk in size with ACN. Polystyrene is completely soluble in ACN or acetone and was shown to shrink in the presence of organic solvents. Polystyrene shrinks in apolar environments from increased electrostatic interactions between the polystyrene polymer layers [21,22]. Hence, the salting-out step displaces polystyrene nanoparticles from water to the ACN phase and leads to osmotic shrinkage. This phenomenon is readily observed with soft polystyrene polymers but seemingly less in hard plastic polymers.

**Figure 5:**
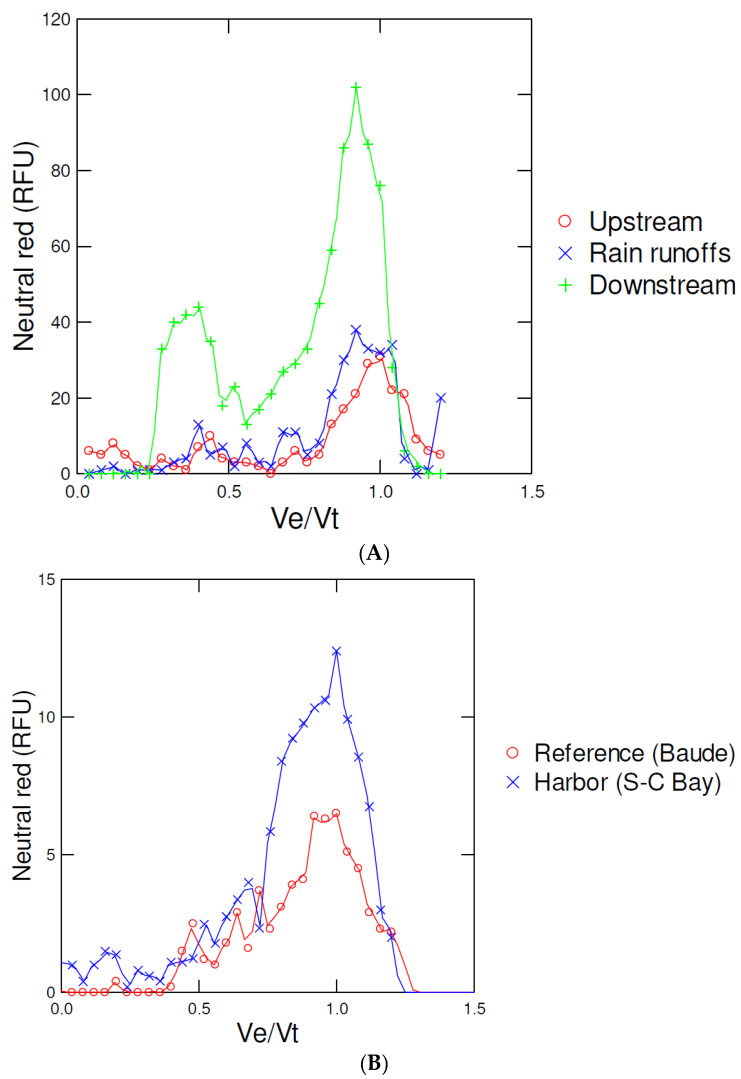
Chromatographic profile of freshwater mussels *Elliptio complanata* caged for 3 months at downstream a large population city (Montréal, Québec, Canada) (**A**) and in wild *Mya arenaria* clams at a polluted harbor, Saint Catherine (S-C) Bay (**B**). Digestive gland homogenates were prepared were extracted using the salting out step and injected to the SEC column using 0.2% Tween-20 and 14 mM NaCl elution buffer. The data represents NR fluorescence at each 1 mL fraction.

This approach was also tested on clam and mussel homogenates at sites under anthropogenic pollution/activity, which are likely sources of plastic pollution [23,24]. The eluted fractions of the Sephacryl S500 column were stained with 10 µM NR dye to detect plastic (apolar) material (Figure 5). In mussels caged for 3 months at downstream and rain runoff sites, NR staining was increased at Ve/Vt = 0.75–0.85 compared to reference Lake values (Figure 5A). In control mussels, the NR peak was Ve/Vt approximately 1 co-eluted with salts, suggesting the presence of other nonpolar low molecular weights compounds soluble in ACN. Moreover, the NR positive and staining peak was also detected at Ve/Vt = 0.3–0.4, suggesting the presence of larger plastic nanomaterials in the 100-nm size range. In *Mya arenaria* clam homogenates collected at a polluted site, the same pattern was observed, i.e., the appearance of NR staining at Ve/Vt = 0.75–0.80 at the polluted site and NR staining at Ve/Vt approximately 1 at the reference site (Figure 5B). Interestingly, the presence of large plastic compounds near the void volume of the column (at 160 nm) was not observed in this case.

The present methodology proposed a convenient and cost-effective means to isolate plastic NPs in tissues based on NR staining. Other, more specific, fluorescent stains could be used, such as molecular rotor probes (9-(2,2-dicyanovinyl)julolidine) that are more specific to plastic materials [12,13]. These approaches could be used as a screening tool to separate and detect plastic NPs but this methodology should be confirmed by more specific methods such as single particle plasma–mass spectrometry or pyrolysis gas chromatography–mass spectrometry [25,26]. The isolated fractions could be further analyzed for metals (e.g., arsenic, copper) or organic compounds to detect *Trojan horse* interactions i.e., xenobiotics sorbed to plastic NPs. They could also be measured using FT–infrared spectroscopy directly or after further ultrafiltration [27]. Nevertheless, the SEC methodology using NR or molecular rotor probes represents a convenient tool for the screening of biological tissues for NPs. However, the proposed methodology is convenient and easy to implement, and more accessible to low-budget laboratories interested in animals exposed to plastic NPs.

## Figures and Tables

**Table 1:** Characteristics of the size exclusion column.

	Ve/Vt ^1^	Comments
Direct injection		
100 nm NP	0.40	NR staining
50 nm NP	0.45	NR staining
Albumin (A280)	0.75	
Protein pool (A280)	0.7–0.95	
NaCl/lipids	0.95–1	Conductivity and NR staining
NaCl/ACN fractionation		
100 nm NP	0.76	Polystyrene osmotic shrinkage
50 nm NP	0.80	Polystyrene osmotic shrinkage
A280 peak	0.9	Not protein origin
NR peak (lipids)	0.95	Phospholipids
NaCl	1	

^1^ Chromatography column: 40 cm × 1 cm (31.4 mL total volume), sample buffer 0.70 M NaCl-0.05% Tween-20 and elution buffer 14 mM NaCl-0.2% Tween-20. Ve: elution volume, Vt: total volume (NaCl). The void volume Vo was Vo/Vt = 0.3 according to the suppliers’ information for Sephacryl S500.

## Data Availability

Supplementary or raw data are available at ECCC upon demand.
